# Mechanisms Responsible for Genetic Hypertension in Schlager BPH/2 Mice

**DOI:** 10.3389/fphys.2019.01311

**Published:** 2019-10-18

**Authors:** Kristy L. Jackson, Geoffrey A. Head, Cindy Gueguen, Emily R. Stevenson, Kyungjoon Lim, Francine Z. Marques

**Affiliations:** ^1^Neuropharmacology Laboratory, Baker Heart and Diabetes Institute, Melbourne, VIC, Australia; ^2^Department of Physiology, Anatomy and Microbiology, School of Life Sciences, La Trobe University, Melbourne, VIC, Australia; ^3^Hypertension Research Laboratory, School of Biological Sciences, Monash University, Clayton, VIC, Australia

**Keywords:** neurogenic hypertension, allopregnanolone, orexin, GABA receptor A, amygdala, hypothalamus, Schlager mice, sympathetic nervous system

## Abstract

It has been 45 years since Gunther Schlager used a cross breeding program in mice to develop inbred strains with high, normal, and low blood pressure (BPH/2, BPN/3, and BPL/1 respectively). Thus, it is timely to gather together the studies that have characterized and explored the mechanisms associated with the hypertension to take stock of exactly what is known and what remains to be determined. Growing evidence supports the notion that the mechanism of hypertension in BPH/2 mice is predominantly neurogenic with some of the early studies showing aberrant brain noradrenaline levels in BPH/2 compared with BPN/3. Analysis of the adrenal gland using microarray suggested an association with the activity of the sympathetic nervous system. Indeed, in support of this, there is a larger depressor response to ganglion blockade, which reduced blood pressure in BPH/2 mice to the same level as BPN/3 mice. Greater renal tyrosine hydroxylase staining and greater renal noradrenaline levels in BPH/2 mice suggest sympathetic hyperinnervation of the kidney. Renal denervation markedly reduced the blood pressure in BPH/2 but not BPN/3 mice, confirming the importance of renal sympathetic nervous activity contributing to the hypertension. Further, there is an important contribution to the hypertension from miR-181a and renal renin in this strain. BPH/2 mice also display greater neuronal activity of amygdalo-hypothalamic cardiovascular regulatory regions. Lesions of the medial nucleus of the amygdala reduced the hypertension in BPH/2 mice and abolished the strain difference in the effect of ganglion blockade, suggesting a sympathetic mechanism. Further studies suggest that aberrant GABAergic inhibition may play a role since BPH/2 mice have low GABA_A_ receptor δ, α4 and β2 subunit mRNA expression in the hypothalamus, which are predominantly involved in promoting tonic neuronal inhibition. Allopregnanolone, an allosteric modulator of GABA_A_ receptors, which increase the expression of these subunits in the amygdala and hypothalamus, is shown to reduce the hypertension and sympathetic nervous system contribution in BPH/2 mice. Thus far, evidence suggests that BPH/2 mice have aberrant GABAergic inhibition, which drives neuronal overactivity within amygdalo-hypothalamic brain regions. This overactivity is responsible for the greater sympathetic contribution to the hypertension in BPH/2 mice, thus making this an ideal model of neurogenic hypertension.

## Introduction

Experimental models of hypertension have been developed over the last half century and have made a major contribution to our understanding of the mechanisms underlying the development and treatment of hypertension. Of particular note here is the spontaneously hypertensive rat (SHR), the stroke-prone SHR from Japan, and other inbred strains such as the Milan (Bianchi et al., 1986) and Lyon strains (Sassolas et al., 1981). There are also a number of transgenic hypertensive rats such as those that have been encoded with the mouse renin gene, which results in hypertension (Sander et al., 1992). By contrast, there have been relatively few hypertensive mouse strains available for hypertension research that have been crossbred from normotensive mice. A frequently studied model is that developed by Gunther Schlager and colleagues in the early 1970s (Schlager, 1974). They established an inbred hypertensive strain of mice (BPH/2, Blood Pressure High) that has subsequently been studied in a wide range of investigations. These have described the phenotype, including genetics (Schlager, 1994; Marques et al., 2011a), cardiovascular function (McGuire et al., 2007; Davern et al., 2009), renal function (Rosenberg et al., 1982), behavior (Elias et al., 1975a), as well as involvement of the sympathetic (Davern et al., 2009; Jackson et al., 2013) and central nervous system (Jackson et al., 2014d). Thus, we have reached a point where we know a great deal about the mechanisms involved in producing the higher blood pressure (BP) in this mouse strain. What has become clear is the important contribution of the sympathetic nervous system (SNS) as well as the renal contribution that appears to involve a specific microRNA regulation of renin (Jackson et al., 2013). Importantly, these revelations are paralleled in the human form of hypertension (Marques et al., 2011c, 2015). Nevertheless, there is still a great deal to discover about these intriguing hypertensive mice. The present review aims to provide an overview of the literature published on BPH/2 mice, with a particular focus on the evidence that these mice represent a unique model of neurogenic hypertension.

## Development and Cardiovascular Characteristics

Genetically hypertensive mice are one of three lines of mice, namely BPL/1 (Blood Pressure Low), BPH/2, and BPN/3 (Blood Pressure Normal), which were concurrently selectively bred based on their BP (Schlager, 1974). It is worth noting that measuring BP in the 1970s in mice involved the tail-cuff technique that required restraining and heating the animals to 37° to determine systolic blood pressure (SBP) (Schlager, 1974). Thus, the target BP for breeding was in effect the level of BP reached during restraint stress, which is known to elevate BP by 30–40 mmHg and is therefore unlikely to be resting BP (Chen et al., 2009). The stable hypertensive phenotype in BPH/2 and hypotensive phenotype in BPL/1 mice were derived through a breeding program applying a two-way selection for high and low SBP respectively, using tail-cuff BP measurements (Schlager, 1974). The third line was randomly selected for breeding from the same base population and resulted in a normotensive (BPN/3) strain of mice. The basis for the cross breeding were eight different inbred strains to produce maximum genetic heterogeneity (C57BL/6J, SJL/J, LP/J, BALB/cJ, RF/J, 129/J, CBA/J, and BDP/J) (Schlager, 1974).

Hypertensive BPH/2 mice display higher BP than control lines from as young as 6 weeks of age, based on tail-cuff measurements (Schlager and Sides, 1997). SBP in BPH/2 mice can range from approximately 100 to 150 mmHg depending on the method of BP measurement (tail-cuff versus radio-telemetry), age (6–23 weeks old), and time of day (light versus dark period) (Schlager and Sides, 1997; Davern et al., 2009). Furthermore, SBP in male and female BPH/2 were found to be similar (Leckie, 2001). Regardless, a statistically and physiologically important and genetically determined difference in BP between the BPN/3 normotensive control mice compared with BPH/2 mice is always apparent. These characteristics give BPH/2 mice an advantage over experimentally or pharmacologically induced hypertensive models, which require extra interventions to initiate the hypertensive phenotype. Further, there can be a large difference between the degree of hypertension in males and females as in the case of Angiotensin II-induced hypertension (Xue et al., 2007).

**Figure 1:**
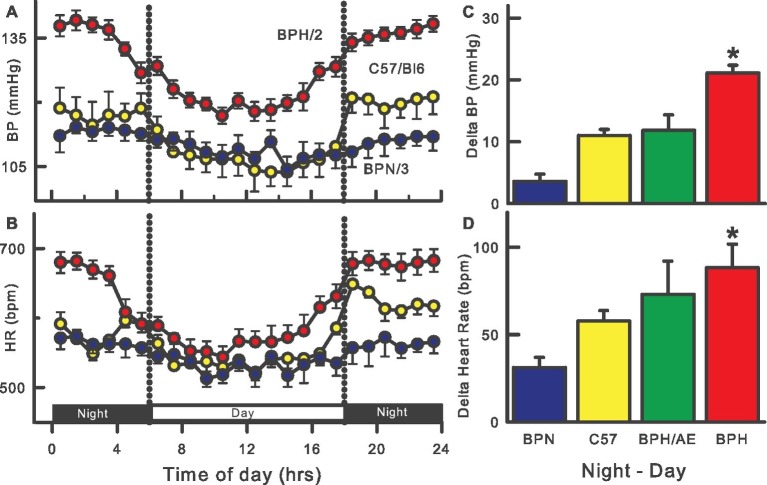
Twenty-four-hour patterns of blood pressure (BP, **A**) and heart rate (HR, **B**) in BPH/2 (red), BPN/3 (blue), and C57/Bl6 (yellow) mice. Right histograms are the average day-night differences for each strain in BP **(C)** and HR **(D)** and also include a BPH/ApoE cross (green). Data adapted from Davern et al. (2009), Jackson et al. (2014b), Al-Sharea et al. (2019). *Indicates *p* < 0.05 between BPN/3 and BPH/2.

The introduction of radio-telemetry measurement for mice was developed in 2000 (Kramer et al., 2000; Butz and Davisson, 2001) nearly a decade after implants for rats (Brockway et al., 1991). Continuous 24-h telemetry measurements confirmed the hypertension in adult BPH/2 compared with BPN/3 mice but the technique was not suitable to measure BP in mice weighing less than 17 g (~6–8 weeks old for BPH/2). At 15 weeks of age, the SBP was 24% and at 23 weeks 18% higher than that observed in BPN/3 mice (Davern et al., 2009). This compares surprisingly well to 24% higher SBP at 14–21 weeks of age as measured 30 years earlier by tail-cuff (Schlager et al., 1979). The BP varies during the 24-h period in mice, following a nocturnal pattern with the highest BP, heart rate (HR), and locomotor activity during the night when the mice are active, and lowest during the day when the mice are less active (Davern et al., 2009). A major characteristic of the BPH/2 strain is a markedly exaggerated day-night difference in BP, HR, and activity compared to the normotensive BPN/3 (Jackson et al., 2014b), C57/Bl6 (Davern et al., 2009), and hypertensive BPH/ApoE mice (Figure 1; Al-Sharea et al., 2019). This day-night difference was small and did not reach statistical significance in the first telemetry study with BPH/2 mice (McGuire et al., 2007) but has been observed in most if not all subsequent studies (Palma-Rigo et al., 2011; Davern et al., 2014; Jackson et al., 2014b,d, 2016; Stevenson et al., 2017; Gueguen et al., 2019; Watson et al., 2019). Reports of HR measured by the tail-cuff technique are variable as to whether BPH/2 mice are tachycardic (Schlager and Sides, 1997) or not (Schlager, 1974) but radio-telemetric measurement of HR under non-stressed conditions consistently shows that BPH/2 mice have higher HR than the normotensive BPN/3 mice (McGuire et al., 2007; Davern et al., 2009).

### Response to Aversive or Non-aversive Stress

Davern and colleagues measured the cardiovascular response to a range of acute stressors in BPH/2 mice compared with BPN/3 mice (Davern et al., 2010b). These included aversive stressors of 5- or 60-min duration such as restraining mice in a plexi-glass tail-cuff restrainer and “shaker stress” which involved placing mice (in cages) on a rotating platform. Mice were also subject to the non-aversive “appetitive” stimulus of being presented with a piece of almond to eat, which produced a pressor response of approximately 20 mmHg, the same magnitude as aversive stressors in BPN/3 mice. The pressor response induced by appetitive stress was only 20% greater in BPH/2 mice whereas aversive stressors were 60–80% greater than in BPN/3 (Davern et al., 2010a,b). The exaggerated cardiovascular response to stress in BPH/2 mice may be due to greater perception of the stress or changes in the central pathways integrating the cardiovascular response. BPH/2 mice appear to be less anxious than controls, based on more frequent entry and greater duration spent in the open arms of an elevated plus/minus maze (Thifault et al., 2001). However, lower activity measurements in BPH/2 mice compared with BPN/3 mice in an open-field test suggest that under certain conditions, BPH/2 mice may be more anxious (Elias and Pentz, 1977). In behavioral terms, based on subjective observation of fighting, BPH/2 mice were reported to be more aggressive than control strains (Elias et al., 1975a). However, BPH/2 mice were shown to be less socially aggressive than control mice through a semi-quantitative assessment of social aggression based on time taken to attack and time spent in non-aggressive and aggressive social interaction toward other mice (Elias et al., 1975a). These inconsistencies in behavioral responses to environmental or socially stressful stimuli could further indicate abnormalities in anxiety and aggression which can be related to hypertension in humans (Perini et al., 1990). Yet the question still remains whether there are any environmental cues in the life of a laboratory mouse that contribute to development of hypertension in this model. This is likely to be a difficult question to answer given that hypertension is reportedly apparent at a very young age, when telemetric BP measurement is difficult to obtain. Furthermore, if stress is driving the hypertension, and since BPH/2 mice spontaneously develop hypertension without any deliberate external interventions such as chronic stress exposure, the stimuli that the BPH/2 mice may perceive as “stressful” would likely be those that are naturally occurring in the life of a laboratory mouse. Furthermore, since BPH/2 and BPN/3 mice are housed under comparable conditions, the same stimuli that lead to hypertension in BPH/2 mice could be considered relatively benign in BPN/3 mice, in that they would not usually lead to hypertension in BPN/3 mice.

## Sympathetic Contribution to the Hypertension in BPH/2 Mice

The earliest indication that BPH/2 mice may have a neurogenic form of hypertension came from findings showing an inverse relationship between whole brain noradrenaline levels and BP in the three lines of mice (BPN/3, BPH/2, and BPL/1) (Schlager et al., 1979). However, dopamine levels in the brain were similar between strains suggesting that the availability of this precursor of noradrenaline was not limiting the production of noradrenaline of BPH/2 mice (Buu et al., 1987). A more discrete assessment of central noradrenaline content revealed lower levels in the midbrain and cerebellum in BPH/2 compared with control mice, despite similar levels of noradrenaline in the hypothalamus and brainstem (Schlager et al., 1983; Buu et al., 1987). As such, strain differences in brain noradrenaline levels are unlikely to be caused by a global abnormality of noradrenaline metabolism because noradrenaline content was not consistently low throughout the whole brain. Further evidence of a central mechanism came from observations that higher brain cytochrome oxidase staining (an indicator of neuronal activity) and greater acute BP response to nicotine occurred in BPH/2 (Schlager and Freeman, 1983; Backer and Schlager, 1984; Denoroy et al., 1985; Strazielle et al., 2004). An important confirmation of the contribution of the SNS to hypertension in BPH/2 mice has only been demonstrated in the last decade, initiated by the finding that ganglion blockade abolished the hypertension in BPH/2 mice, ultimately reducing BP to levels comparable with normotensive BPN/3 control mice (Davern et al., 2009). Furthermore, even when the depressor response to ganglion blockade was represented as a percentage of baseline, the response was still greater in BPH/2 compared with BPN/3 normotensive controls (Davern et al., 2009), unlike findings in SHR where the percentage reduction by hexamethonium was the same as the control normotensive strain (Touw et al., 1980). A similar 23% reduction in BP was observed with the central sympatholytic drug α-methyldopa in both SHR and normotensive Wistar rats (Head and de Jong, 1986). Thus, the BPH/2 differs from the SHR in being more dependent on the SNS for hypertension. Greater SNS activity in BPH/2 was also supported by a greater mid-frequency BP power, which is an indirect indicator of vasomotor SNS activity, in BPH/2 compared with BPN/3 mice (Davern et al., 2009). Using ganglion blockade with prior angiotensin-converting-enzyme (ACE) inhibitor pretreatment, the SNS contribution to BP was 1.7-fold greater in BPH/2 mice compared with BPN/3 mice during both light (inactive) and dark (active) periods (Jackson et al., 2013). Importantly, the contribution of the SNS to BP did show typical circadian fluctuations, with the SNS excitation peaking during the active period in both strains. This suggests that the cause of the hypertension is likely to be 24-h overactivity of the SNS (tonic component) but there is still an overlying circadian pattern with the SNS activity being greatest (in all mice) during the active period (phasic component). Greater tyrosine hydroxylase staining in the kidney of BPH/2 mice also indicated sympathetic hyperinnervation (Jackson et al., 2013) which has been seen in other models of hypertension such as SHR (Cassis et al., 1985). More recently, renal noradrenaline levels were reported to be markedly greater in BPH/2 compared with BPN/3 mice and bilateral renal denervation was shown to reduce the hypertension by one-third in BPH/2 mice without affecting BP in BPN/3 mice (Gueguen et al., 2019). Interestingly, the hypotensive response may also involve the renin-angiotensin system (RAS) since the enhanced renin mRNA levels in BPH/2 mice were normalized following renal denervation (Gueguen et al., 2019). Overall, greater sympathetic drive to the kidneys and possibly more generally, contribute largely to the hypertension in BPH/2 mice.

## Brain Regions Involved in Mediating the Hypertension in BPH/2 Mice

### Amygdalo-Hypothalamic Pathway Contribution to Hypertension in BPH/2 Mice

The amygdala integrates sensory (e.g., olfactory) and cognitive information to initiate behavioral responses to specific stimuli during aversive psychological and physiological stress as well as reproductive stimuli. In addition, a major pathway for sympathetic activation involves projections from the amygdala to the anterior hypothalamus, the dorsomedial hypothalamus (DMH), and the ventromedial hypothalamus (VMH) (Choi et al., 2005). These projections are glutamatergic and GABAergic (Choi et al., 2005). Davern and Head suggested that forebrain regions including the medial amygdala (MeAm) may be responsible for driving the hypertension in BPH/2 mice (Davern and Head, 2011). Indeed, marked differences in neuronal activity were observed between BPH/2 and BPN/3 mice in different brain regions before and after the surge in BP that occurs during the onset of the dark (active) period. In particular, Davern and colleagues showed that the number of neurons expressing c-Fos, a protein marker of recent neuronal activation (Li and Dampney, 1992), in the DMH, the paraventricular hypothalamus (PVN), the central amygdala, and the MeAm of BPH/2 mice was markedly greater compared with BPN/3 mice (Davern et al., 2009). Interestingly, the authors reported that the MeAm was the only region to display greater c-Fos stained neurons in both the light (inactive) and dark (active) periods in BPH/2 compared with BPN/3 mice. Furthermore, neuronal activity within the MeAm of BPN/3 and BPH/2 mice strongly correlated with BP and also with the decrease in BP caused by ganglion blockade (Davern et al., 2009). Taken together, these findings suggest that greater neuronal activity in the MeAm of BPH/2 mice may lead to elevations in BP *via* influences on the SNS.

The MeAm was also one of the brain regions with greater c-Fos containing neurons in BPH/2 mice compared with BPN/3 mice following exposure to 1 h of dirty cage switch stress (Davern et al., 2010a). This involved placing a mouse in a previously occupied cage and after an hour, greater c-Fos stained neurons were observed in the CeAm, DMH, PVN, and rostro-ventrolateral medulla (RVLM) (Davern et al., 2010a). Using c-Fos-immunohistochemistry, the MeAm can be readily observed as playing a major role in regulating stress (Cullinan et al., 1995; Beckett et al., 1997; Dayas et al., 2001; Dielenberg et al., 2001; Porter and Hayward, 2011). Moreover, this region is particularly important for integration of olfactory and chemosensory signaling involved in predator and territorial responses and reproduction (Dielenberg et al., 2001; Samuelsen and Meredith, 2009). Although Dielenberg and colleagues identified that predominantly psychological stressors activated the MeAm, it was interesting to note that physiological stressors such as hemorrhage also selectively activated the CeAm (Dielenberg et al., 2001). However, greater levels of c-Fos-staining is not the only indication that the MeAm is involved in regulating the response to stress. Many studies using lesions, electrical or pharmacological stimulation or inhibition have supported the view that the MeAm plays a key role in mediating the cardiovascular, hormonal, and behavioral responses to stress (Han et al., 1996; Kubo et al., 2004; Herdade et al., 2006; Fortaleza et al., 2009, 2012; Solomon et al., 2010; Vinkers et al., 2010).

#### Effect of Lesions of the Medial Amygdala

The idea that the amygdala contributes to hypertension is not new and was shown to be important to the development of hypertension in SHR, based on the antihypertensive effect of electrolytic lesions of the amygdala (Folkow et al., 1982). Fukumori and colleagues showed that the MeAm region in particular influences hypertension in SHR as excitotoxic lesions created in the MeAm of SHR at 4 weeks of age attenuated the development of hypertension by 14–16 weeks of age (Fukumori et al., 2004). A normotensive strain was not included as a control in this study, and so drawing conclusions about the contribution of the MeAm to hypertension as opposed to BP maintenance is problematic.

Microinjections into the MeAm with ibotenic acid, which ablates cell bodies but not fibers of passage, resulted in a hypotensive effect that abolished 64% of the hypertension in BPH/2 but had no effect in BPN/3 (Jackson et al., 2014d). MeAm lesions also decreased the depressor response to ganglion blockade as well as the mid-frequency BP power in BPH/2 mice, suggesting the lesions had a sympatholytic effect. The hypotensive effect of the lesions was similar during the dark (active) and light (inactive) periods suggesting that the MeAm has a tonic influence on BP in BPH/2 but not in normotensive animals. Intriguingly, despite MeAm lesions lowering BP in BPH/2 mice, lesions had little effect on the exaggerated cardiovascular response to stressors in these mice, suggesting that established hypertension is independent of the exaggerated cardiovascular response to stress.

### Rostro-Ventrolateral Medulla Contribution to Hypertension in BPH/2 Mice

Premotor sympathetic neurons within the RVLM are important for regulating sympathetic vasomotor tone (Dampney et al., 1982; Horiuchi and Dampney, 1998) and have been implicated in many models of hypertension including renovascular, Goldblatt, L-NAME–induced, and SHR models (Muratani et al., 1993; Bergamaschi et al., 1995, 1999; Matsuura et al., 2002). Intriguingly, assessment of two different markers of neuronal activity, c-Fos and cytochrome oxidase staining, indicated that RVLM neuronal activity was similar in BPN/3 and BPH/2 mice (Strazielle et al., 2004; Davern et al., 2009). SHRs are known to be responsive to centrally acting sympatholytic drugs such as rilmenidine, which act predominantly at the level of the RVLM (Sannajust et al., 1989; Mayorov et al., 2001; Zhang and Abdel-Rahman, 2002). A comprehensive study administering rilmenidine to BPH/2 mice *via* multiple routes (i.p., i.c.v., s.c., p.o.) and for a range of durations (30 min to 2 weeks) revealed that the sympathetic vasomotor inhibitory effect of rilmenidine is minimal and similar in hypertensive BPH/2 and normotensive BPN/3 mice (Jackson et al., 2014c). Rilmenidine administered acutely (i.p.) did cause a greater depressor and marked bradycardic effect in BPH/2 compared with BPN/3 mice. By contrast, pre-treatment with atropine (a muscarinic receptor antagonist) prior to rilmenidine actually abolished any difference between the two strains, ultimately revealing that the difference between them was due to vagal excitatory effects of rilmenidine, and the sympatholytic effects were comparable in BPH/2 and BPN/3 mice (Jackson et al., 2014c). Furthermore, chronic administration of rilmenidine, where the vagal excitatory effects are known to be less prominent (Godwin et al., 1998; Parkin et al., 2003), showed only minor and similar reductions in BP between the strains (Jackson et al., 2014c). Together, the lack of neuronal activity differences between strains and lack of responsiveness to rilmenidine suggest that the RVLM is not a major driver of the sympathetically mediated hypertension in BPH/2 mice. Thus, if the RVLM, which is a major premotor sympatho-regulatory brain region, is not playing a major role in hypertension, it is possible that the greater sympathetic outflow may be mediated *via* sympathetic premotor neurons other than those located in the RVLM. In this context, the PVN, which is a region that contains sympathetic premotor neurons that project directly to sympathetic preganglionic neurons in the spinal cord (Strack et al., 1989), could be a nucleus of interest as it has been shown to be highly activated during the dark active period in BPH/2 mice (Davern et al., 2009).

## Molecular Contributions to Hypertension in BPH/2 Mice

Wright and colleagues performed the first genome-wide scan in F2 intercrosses of BPH/2 and BPL/1 mice as well as backcrosses revealed significant linkage on chromosome 10 and 13 with suggestive linkages on chromosomes 2, 6, 8, and 18. Exactly what micro-satellite polymorphisms are distinguishing high- and low-BP mice has not as yet been discovered (Wright et al., 1999). The earliest examination using genome-wide microarray analysis from adrenal tissue was designed to identify genes whose difference in expression contributes to hypertension in the Schlager BPH/2 mice and used the BPL/1 strain for comparison rather than the normotensive strain (Fries et al., 2004). This may be an issue if the genes differently expressed in the low-BP strain compared to normotensive mice are different to those differentially expressed in the strain with high BP. It should also be remembered that 1) the differential expression of particular genes may contribute directly to the condition, or 2) that differential expression may be a secondary response to a primary difference in expression of other gene(s), or 3) the differential expression may be a compensatory response to the BP elevation or another phenotypic difference between each mouse strain. Using a systems biology approach, Fries and colleagues found differences in pathways associated with the SNS activity, oxidative stress, and also carbohydrate metabolism (Fries et al., 2004). A later comparison of BPH/2 to SHR showed that the concentration of enzymes responsible for noradrenaline and adrenaline synthesis, tyrosine hydroxylase, and phenylethanolamine N-methyltransferase were greater in BPH/2 mice but normal in SHR. This suggests species differences in the contribution of the SNS to the hypertension. This is, however, consistent with the differential effects of ganglionic blockade mentioned earlier. Specifically, this treatment abolished the difference in BP between the BPH/2 and BPN/3, but in rats, the difference in BP between SHR and the normotensive rats remained after ganglion blockade. By contrast, the activity of oxidative stress pathways is elevated in both hypertensive strains of the two species (Friese et al., 2005). More recently, an integrated combination of transcriptomics, bioinformatics, and molecular biology of the BPH/2 compared to the BPL/1 strain led the investigators to suggest that from the differential expression analysis, *HOXA3*, *SRY,* and transcriptional factor *Yy1* might predict BP in humans. Further analysis of human population indicated that a single nucleotide polymorphism of *Yy1* was associated with BP, body mass index, and fasting glucose. They suggested this gene as the strongest candidate influencing hypertension and metabolic syndrome (Friese et al., 2012).

Puig and colleagues examined liver, heart, kidney, and vessels from all three of the Schlager strains using microarray analysis of 38,000 transcripts (Puig et al., 2010). A number of genes known to be involved in BP regulation were differentially expressed between the strains, one of these genes being the natriuretic peptide receptor gene *Npr1*. Since expression of *Npr1* is upregulated in hypertension, it is likely countering the effects of hypertension (Puig et al., 2010). The chemokine receptor gene *Ccr5* and the gene for arachidonic acid metabolic enzyme *Cyp2j2* were also found to be differentially expressed and have been implicated in hypertension (Puig et al., 2010). A later microarray analysis of kidney tissue by Chiu and colleagues compared expression in male and female BPH/2 with male and female normotensive BPN/3 (Chiu et al., 2014). Several genes were differentially expressed and associated with hypertension. One was angiopoietin-like 7, which may reflect increased vascular pressure in the kidney. Others were *Hdc* and *Cndp2,* which are associated with histamine metabolism and possibly sympathetic activity or inflammation (Chiu et al., 2014). A gene associated with vascular aging, *Edn3*, was also differentially expressed as were the DNA maintenance genes, *Mcm6 and Dna2*, which are involved in telomere length maintenance. A further analysis revealed that BPH/2 mice have shorter telomeres, but this occurs after hypertension is established and is a consequence rather than a cause of the hypertension (Chiu et al., 2016). Other bio-informatic analyses of the Chiu et al. dataset have been made by Gao et al. (2017) and further novel uses of meta-genome data have suggested transcriptome networks associated with hypertension, so providing scope for new therapeutic targets (Zubcevic et al., 2017). A more recent transcriptome analysis of cardiac endothelial cells suggested that differences between prehypertensive and hypertensive adult BPH/2 mice are related to fibrosis (Nelson et al., 2018). While endothelial dysfunction is known in the BPH/2 strain, no differences in the endothelial genes associated with the nitric oxide pathway were detected. However, expression of the PPARα pathway genes was reduced. These genes encode proteins thought to be protective against the damage from hypertension-induced oxidative stress (Nelson et al., 2018). Interestingly, the authors found that treating BPH/2 mice with antihypertensive drugs such as losartan or the calcium channel blocker amlodipine reversed the expression pattern of some genes but not others. Such interventions should help discriminate between the effect of hypertension itself on gene expression as opposed to effects arising from strain differences.

While the earlier microarray studies of Fries and colleagues suggested a contribution of the SNS to the hypertension in the BPH/2 mice, most other studies concentrated on peripheral organs such as adrenal, liver, blood vessels, heart, and kidney. While the kidney is well known to contribute to a number of forms of hypertension, it is surprising that there have been few studies examining the central nervous system (CNS). A major study by Marques and colleagues used microarray and quantitative real-time polymerase chain reaction (qPCR) of the hypothalamus of young and adult BPH/2 and BPN/3 mice (Marques et al., 2011a). This work revealed an elevation in the expression of genes implicated in oxidative stress and inflammation in BPH/2 mice as well as higher expression of hypocretin (Orexin) and the neuropeptide S receptor (Npsr1). Hypocretin neurons display synaptic plasticity in response to overnight fasting (Horvath and Gao, 2005) while Npsr1 administration can reduce the anxiety-like behavior in mice associated with open field, elevated plus maze, light-dark box, and marble burying (Okamura and Reinscheid, 2007). A further study of the genes that might contribute to the exaggerated nocturnal differences in BP in BPH/2 was conducted and revealed 212 differentially expressed genes including those for vasopressin, oxytocin, and thyrotropin-releasing hormone (Marques et al., 2011b). Although not highlighted in these analyses, there were also differences in expression of a number of type A gamma-aminobutyric acid (GABA) receptor (GABA_A_R) subunits in the hypothalamus of BPH/2 compared to BPN/3 (Marques et al., 2011a,b). This has prompted further research into the possible contribution of GABA_A_R to neurogenic hypertension – in particular in the BPH/2 strain (Davern et al., 2014).

## Neuropeptide and Neurosteroid Contribution to Hypertension in BPH/2 Mice

### Gamma-Aminobutyric Acid Receptors

**Figure 2:**
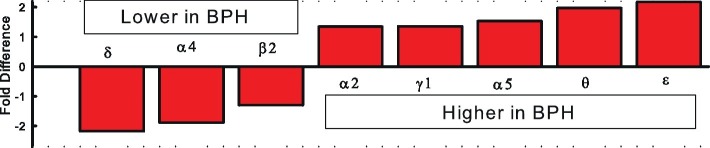
Fold differences in GABA_A_R subunit expression in the hypothalamus of BPH/2 compared to BPN/3 mice (Marques et al., 2011a,b).

The GABA_A_R is a heteromeric pentamer made up of five of 19 currently known subunits arranged in a combination of two α, two β, and a third (γ, δ, ϵ, ρ, or θ) subunit all arranged around a chloride conducting central pore. The combination of subunits gives rise to the vast number of structurally unique GABA_A_R, which contributes to its diverse functionality. The amygdalo-hypothalamic pathway contributes to hypertension in both BPH/2 mice and SHR and may involve a common mechanism leading to increased sympathetic vasomotor tone. Combining the microarray finding of altered GABA_A_R subunit expression in the hypothalamus with the lower levels of GABA_A_R in the hypothalamus and amygdala of SHR (Kunkler and Hwang, 1995), we suggested that inadequate GABAergic inhibitory signaling may contribute to the higher activity in the pre-sympathetic pathways leading to the hypertension in BPH/2 (Davern et al., 2014). A lack of GABAergic inhibition of the MeAm in BPH/2 mice would be expected to result in tonic hyperactivity of these neurons and activation of hypothalamic autonomic influences. Patterns of expression of the different subunits of GABA_A_R indicate that the δ, α4, β2 subunits are underexpressed in BPH/2 hypothalamus while α2, γ1, α5, θ and ε subunits are more highly expressed in BPH/2 compared to BPN/3 (Figure 2; Marques et al., 2011a).

#### Effect of the Benzodiazepine Diazepam

It is well known that benzodiazepines such as diazepam can modulate GABA_A_R activity by binding to an allosteric site on the receptor (Bormann, 1991). Chronic administration of diazepam in drinking water reduced BP of the BPN/3 mice but had no effect on the BP of BPH/2 mice (Davern et al., 2014). We used triple labeling immunohistochemistry to show that neurons in the hypothalamus and amygdala of diazepam-treated BPH/2 mice that had been activated by restraint stress (contained c-Fos protein in the nucleus) also contain GABA_A_R. By contrast, neurons activated by restraint stress in the same regions of diazepam-treated BPN/3 mice did not label for GABA_A_R. Presumably this is because they were inhibited by the diazepam treatment unlike those of BPH/2 mice, which did not respond to diazepam. The inability of diazepam to be effective in BPH/2 may have allowed greater activity within the neurons of the amygdala and the hypothalamus (Davern et al., 2014). Thus, there appears to be a fundamental difference between the GABA_A_R in the two strains that respond to benzodiazepines. The benzodiazepine binding socket in the GABA_A_R involves the α1, α2, α3, α5, and γ2 subunits (Benson et al., 1998) and, of these, only the α2 subunit was differentially expressed between BPH/2 (higher) and BPN/3 (lower expression).

#### Effect of the Neurosteroid Allopregnanolone on BPH Hypertension

**Figure 3:**
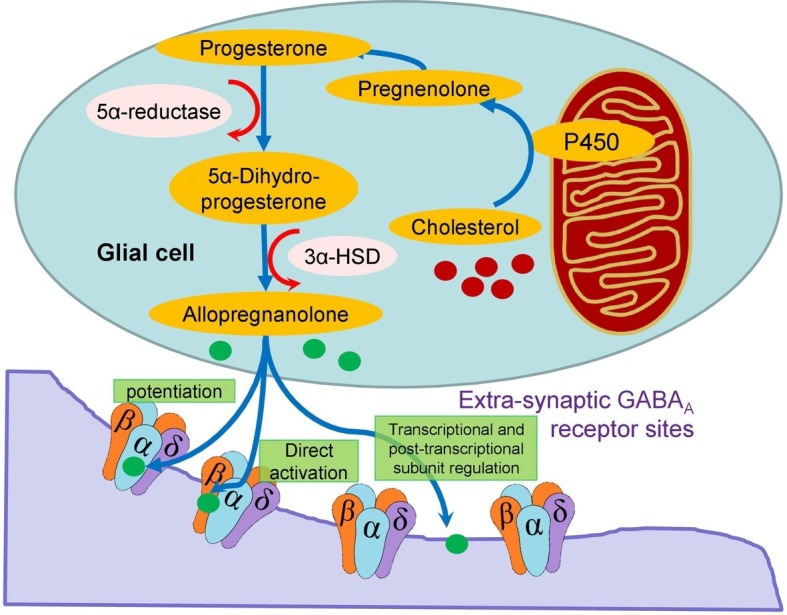
Schema showing the intracellular synthesis of allopregnanolone in glia from cholesterol and the direct and indirect action of the neurosteroid on extracellular GABA_A_R. The latter involves potentiation and transcription and post-transcriptional subunit regulation.

The reduced expression of the δ, α4, and β2 subunits in the hypothalamus of the BPH/2 compared with BPN/3 mice (Figure 2) is a key finding as these are also the same combination of subunits common to extrasynaptic GABA_A_R (Smith et al., 2007) that are responsible for tonic GABAergic inhibition in the brain (Farrant and Nusser, 2005). In the rat thalamus, all δ subunits co-exist with an α4 subunit (Sur et al., 1999). The δ, α4, and β2-GABA_A_R have a high affinity for GABA, which means they sense its ambient levels of GABA in the extrasynaptic space and provide a background level of inhibition (Farrant and Nusser, 2005). These receptors are also more resistant to desensitization (Farrant and Nusser, 2005). The δ, α4, and β2-GABA_A_R are the most sensitive to modulation from allopregnanolone-like neurosteroids. Allopregnanolone can be synthesized in glia in the CNS from cholesterol *via* a pathway involving initial transport to the mitochondria and conversion by cytochrome P450 to pregnenolone, which is converted to progesterone and 5α-dihydroprogesterone and finally to allopregnanolone (Figure 3; Farrant and Nusser, 2005). The effect of allopregnanolone on δ-GABA_A_R is an allosteric modulation of chloride currents by enhancing chloride channel opening time and potentiating inhibition (Hosie et al., 2006). There is potentiation and direct activation (Figure 3). Most critically, treatment with allopregnanolone or its precursor, progesterone, can increase the expression of the exact subunits that are reduced in BPH/2, namely the α4 and δ subunits, through transcriptional and post-transcription effects (Figure 3; Gulinello et al., 2001). Thus, treatment with allopregnanolone may alter subunit composition of GABA_A_R in a manner that promotes GABAergic inhibition and thus increases tonic inhibition in the brain. Patients and animal models of chronic stress, anxiety disorders, or depression have lower levels of allopregnanolone than controls (Serra et al., 2000; Rasmusson et al., 2006).

Thus, we hypothesized that the lower levels of expression of the δ, β2, and α4 subunits of GABA_A_R in BPH/2 may provide insufficient tonic inhibition of sympatho-excitatory neurons in the amygdala and hypothalamus and therefore these hypertensive mice might respond to allopregnanolone. Treatment of adult Schlager mice with 200 μg/kg/h of allopregnanolone (s.c.) reduced BP by 40% in BPH/2 after 14 days with no effect in BPN/3 mice (Stevenson et al., 2017). Lower doses of allopregnanolone and shorter treatment duration (1 week) were ineffective on BPH/2 hypertension suggesting that transcriptional changes to subunit composition of GABA_A_R were required rather than a direct activation or potentiating of the neurosteroid (Stevenson et al., 2017). We also observed a reduction in the depressor response to pentolinium in BPH/2 and not in BPN/3, indicating less sympathetic contribution to BP. Allopregnanolone had no effect on mouse activity or anxiety levels suggesting that the BP changes are not secondary to behavioral effects (Stevenson et al., 2017). The low dose suggests that the particular GABA_A_R involved has a high affinity for allopregnanolone with effective doses well below those that induce sedation or even anxiolysis (Deo et al., 2010). This is consistent with the high affinity characteristics of the extra-synaptic δ-containing GABA_A_R. Both the pressor response to dirty cage swap stress and the elevation in neuronal activity observed in the MeAm and PVN of BPH/2 mice were reduced or abolished following treatment with allopregnanolone (Stevenson et al., 2017). These findings suggest that the MeAm and the PVN are likely major sites of action of allopregnanolone. While we observed a substantial reduction in BP in the BPH/2 mice, the hypertension was not abolished, suggesting other mechanisms may also contribute to the elevated BP in these mice.

### Orexin

Orexin is a neuropeptide known for its role in regulating feeding, stress, and arousal but importantly it is also able to regulate BP, HR, and sympathetic activity (Sakurai, 2005; Carrive, 2013; Inutsuka and Yamanaka, 2013). Orexin/hypocretin was initially identified as a potential contributor to the hypertension in BPH/2 mice based on a study showing that BPH/2 mice have at least 2-fold higher *hcrt* mRNA in the hypothalamus than BPN/3 mice (Marques et al., 2011a). Furthermore, BPH/2 mice were shown to have almost 30% more orexinergic neurons than BPN/3 mice (Jackson et al., 2016). To determine whether this augmented orexin level actually contributes to the hypertension in BPH/2 mice, almorexant, a dual orexin receptor antagonist, was administered to BPH/2 mice (Jackson et al., 2016). Almorexant caused a marked reduction in BP in BPH/2 mice during the dark (active) period but had no effect in the BPN/3 mice, whereas during the light (inactive) period, there was no reduction from baseline in either strain (Jackson et al., 2016). While a portion of the hypotensive effect of almorexant was due to a concurrent reduction in locomotor activity, part of the hypertension could still be attributed to an activity-independent reduction in BP (Jackson et al., 2016). Furthermore, the reduction in BP during the dark period in BPH/2 mice was associated with a reduction in depressor response to ganglion blockade and mid-frequency BP power, both suggesting almorexant reduced SNS activity. Orexin is known to contribute to hypertension in at least two other models of hypertension including SHR and stress-induced hypertension, but does not appear to regulate basal BP maintenance in normotensive animals (Lee et al., 2013; Li et al., 2013; Xiao et al., 2013).

### Renin-Angiotensin System in BPH/2 Mice

The renin-angiotensin system (RAS) has been explored in a variety of ways in BPH/2 mice including genetic investigations, analysis of expression or activity of RAS components, and pharmacological assessment of the RAS contribution to BP. Yet, delineating the contribution of the RAS to the hypertension in BPH/2 mice has proven quite complex. Angiotensin II (AngII) is an important effector hormone, being part of the RAS, which is well known to contribute to BP regulation by influencing dipsogenic and sodium appetitive responses, vasopressin release, and importantly sympathetic vasomotor tone (Keil et al., 1975; Head, 1996; Fitzsimons, 1998). In 2001, Leckie described a larger depressor effect of the ACE inhibitor captopril in BPH/2 mice (Leckie, 2001), but plasma renin levels and renin enzyme activity were reportedly similar among BPN/3, BPH/2, and BPL/1 mice (Iwao et al., 1984). However, there are conflicting findings with regard to renin activity in the submandibular glands of BPH/2 mice, regarding whether it is comparable with BPN/3 mice or greater in BPH/2 mice (Iwao et al., 1984; Uddin et al., 2003a; Uddin and Harris-Nelson, 2004). Additionally, lower angiotensin I in the submandibular gland is hypothesized to reflect a faster rate of conversion to AngII but this has not been verified (Uddin and Harris-Nelson, 2004). Gene array of the liver, heart, and aorta reveals no strain difference in expression of any major components of the RAS (Puig et al., 2010). However, there is a 1.3-fold greater expression of ACE specifically in the kidney of BPH/2 mice compared with BPN/3 mice, suggesting tissue-specific RAS differences may be apparent (Puig et al., 2010). The differences in expression or activity of the RAS could be due to differences in the state of the animal immediately prior to collection of tissue, as RAS activity is subject to changes in state such as sleep, wakefulness, and stress (Jindra and Kvetnansky, 1982; Brandenberger et al., 1994).

Using a large dose of losartan for 14 days [150 mg/kg/day, angiotensin II type 1 receptor (AT_1_R) antagonist], Palma-Rigo and colleagues found little difference between the hypertension induced by the drug in both strains, which was in the order of 23–25 mmHg (Palma-Rigo et al., 2011). This study assessed potential interaction between the SNS and the RAS in BPH/2 mice, whereby the effect of ganglion blockade was assessed before and during chronic AT_1_R inhibition (Palma-Rigo et al., 2011). The findings suggest that there was no interaction between the RAS and SNS in BPH/2 mice that might contribute to the hypertension. However, high doses of losartan were administered, which likely inhibited both the central and peripheral RAS. If the central and peripheral RAS contributions to hypertension of BPH/2 were in opposite directions, the high dose of losartan that blocked both would not necessarily reveal these subtle differences.

#### Contribution From Renal Renin-Angiotensin System and Influence of MicroRNA-181a

When an acute treatment with the ACE inhibitor enalaprilat was given in the active period, it produced a small hypotensive effect in BPH/2 but no effect in BPN/3 (Jackson et al., 2013). One limitation of using an ACE inhibitor to assess the contribution of AngII to hypertension in BPH/2 mice is that ACE inhibitors also reduce the degradation of bradykinin, which itself could have a vasodilatory effect. However, the vasorelaxation caused by bradykinin was previously shown to be markedly reduced in BPH/2 compared with BPN/3 mice (McGuire et al., 2007). During the active period when enalaprilat produced hypotension, there was also a 1.6-fold greater expression of the renin gene and lesser abundance of the microRNA microRNA-181a (miR-181a). Interestingly, the hypotension from enalaprilat and the difference in renin and miR-181a expression were not observed during the inactive period (Jackson et al., 2013). MiR-181a has been shown previously to bind to the 3′ untranslated region and to regulate renin mRNA in humans and is reciprocally expressed with respect to renin in kidneys of hypertensive subjects compared to normotensive mice (Marques et al., 2011c, 2015). Thus, when renin expression levels are high in hypertensive compared to normotensive mice, miR-181a levels are the opposite (Marques et al., 2015). Likewise, in BPH/2 mice, miR-181a was downregulated specifically during the active period in BPH/2 mice, corresponding to the time when RAS activity was greatest. While miR-181a negatively regulates renin mRNA levels, it is unknown what regulates miR-181a. The possibility that miR-181a might be regulated by the SNS was suggested by a correlation between the depressor response to ganglion blockade with renal abundance of miR-181a (Jackson et al., 2013). BPH/2 mice also have greater renal sympathetic innervation density as identified by tyrosine hydroxylase staining of cortical tubules (Jackson et al., 2013) which is where in human, miR-181a is expressed (Marques et al., 2015). Also, renal denervation reduced the higher renal expression of the renin gene and lowered renal renin levels but had no effect on plasma renin concentrations (Gueguen et al., 2019). In a preliminary abstract report, BPH/2 mice treated with a miR-181a mimic had a reduced level of hypertension and normalized levels of renal renin expression (Jackson et al., 2014a). Injection of such miR-181a mimetics *in vivo* has previously been effectively used to restore low levels of miR-181b in the vasculature of mice (Sun et al., 2012).

The mechanism by which peripheral AngII increases BP in BPH/2 mice has not been assessed and could be produced by anti-natriuretic or vasoconstrictive effects. Alternatively, circulating AngII could even potentially facilitate the augmented SNS in BPH/2 mice. However, the finding that pretreatment with an ACE inhibitor did not attenuate the exaggerated depressor response to ganglion blockade suggests that there is no overt facilitation of the SNS by the peripheral RAS in BPH/2 mice (Jackson et al., 2013). Direct vasoconstrictive effects of AngII are likely to contribute to elevated BP in BPH/2 mice. This is supported by a preliminary abstract report showing that AngII causes greater vasoconstriction in BPH/2 mice using myography (Jelinic et al., 2018). It is also possible that renal sympathetic hyperinnervation and increased AngII levels result in increased anti-natriuretic effects in BPH/2 mice. Rosenberg and colleagues measured glomerular filtration rate in mature BPH/2 mice and while it tended to be lower than in BPN/3 mice, this did not reach significance (Rosenberg et al., 1985). Furthermore, BPH/2 mice are reportedly not salt sensitive (Leckie, 2001). Total body water as a percentage of body weight is comparable in BPH/2 and BPN/3 mice, indicating that volume expansion due to greater retention of water was unlikely to contribute to the hypertension (Jackson et al., 2014b). Thus, greater anti-natriuretic effects of RSNA or AngII do not seem to be likely mechanisms causing hypertension in BPH/2 mice.

#### Central Renin-Angiotensin System Contribution to Hypertension in BPH/2

In addition to the peripheral RAS, a separate renin-angiotensin system within the CNS has been reported to contribute to the hypertension of animal models including SHR, phenol renal injury hypertension model, and cold-induced hypertension (Huang and Leenen, 1996; Ito et al., 2002; Sun et al., 2002; Ye et al., 2002). Circulating AngII can affect the CNS by acting on AT_1_R in areas such as the circumventricular organs that do not have a blood-brain barrier. Since it has been suggested that there is an apparent overactivity of the peripheral RAS, which was based on the greater acute depressor response to ACE inhibition (Jackson et al., 2013), assessment of the role of the central RAS was pertinent. Gene array analysis of the hypothalamus in BPH/2 mice did not identify any major differences in the expression of components of the brain RAS, to suggest that there are inherent abnormalities in BPH/2 mice compared with BPN/3 controls (Marques et al., 2011a,b). However, this does not necessarily refute the possibility that central RAS signaling could contribute to hypertension in BPH/2 mice, as is the case in other models of hypertension (Muratani et al., 1993; Campese et al., 2000).

The suggestion that augmented central AngII stimulation of AT_1_R may contribute to the hypertension in BPH/2 mice was recently examined. Surprisingly, the activity of central AT_1_Rs appears to contribute equally or less to BP regulation in BPH/2 mice (Jackson et al., 2018). This was based on a study which administered AT_1_R antagonists (i.c.v.) both acutely and chronically in BPH/2 and BPN/3 mice. Acute administration of candesartan actually produced a lesser depressor response in BPH/2 mice compared with BPN/3 mice during the dark (active) period, whereas the response was comparable between strains in the light (inactive) period (Jackson et al., 2018). Subcutaneously infused losartan for 2 weeks produced the same small hypotensive effect as centrally infused losartan, suggesting that the central AT_1_R contribution to BP is not evident in BPH/2 mice. Reactive oxygen species (ROS) such as superoxide can be second messengers for AngII-mediated signaling in the brain (Zimmerman et al., 2002; Chan et al., 2005); therefore, the contribution of central ROS to hypertension in BPH/2 mice was also assessed. When the ROS scavenger tempol and the superoxide dismutase (SOD) mimetic resveratrol were administered (i.c.v.), the acute cardiovascular response was comparable between BPN/3 and BPH/2 mice, suggesting that central ROS is unlikely to play a role in the hypertension (Jackson et al., 2018). Taken together, these findings indicate that a generalized overactivity of the central AT_1_R-ROS signaling is not driving the hypertension in BPH/2 mice. However, due to the intricacies of AngII signaling in the brain, this study cannot completely exclude a role for the central RAS in discrete regions, which would require a more targeted approach. It is interesting to note that the greater contribution of the peripheral RAS distinctly contrasts the lesser contribution of central AT_1_R activity to hypertension in BPH/2 mice during this dark (active) period (Jackson et al., 2013, 2018). Exposure to high levels of circulating AngII in rabbits for longer than a few days has previously been shown to result in desensitization of neurons to AngII activation in circumventricular organs and sensitization in hypothalamic regions (Davern and Head, 2007). Furthermore, circulating AngII is also shown to influence brain AT_1_R expression, in a site-specific manner (Thomas and Sernia, 1985). It would be interesting to investigate whether greater circulating AngII in BPH/2 mice could also potentially lead to downregulation of AT_1_Rs centrally, which may account for the smaller depressor response induced by candesartan (i.c.v.) in BPH/2 mice. Nonetheless, it seems that in contrast to the peripheral RAS, overactivity of the central RAS does not contribute to the hypertension in BPH/2 mice.

## Heart, Vessel, and Kidney Structure and Function

Despite being hypertensive from a relatively young age, BPH/2 mice do not have greater absolute heart weight nor left ventricle weight compared with normotensive BPN/3 mice (Schlager et al., 1979; Schlager and Sides, 1997). However, the body weight of BPH/2 mice was lower than BPN/3 mice, so when expressed as HW:BW ratio, there was a tendency for this measure to be greater in BPH/2 mice (Schlager and Sides, 1997) and normotensive C57Bl/6 mice (Elias et al., 1975b). Thus, a degree of cardiac hypertrophy is apparent in BPH/2 mice but it appears to be mild. Interestingly, a later transcriptome analysis of cardiac endothelial cells revealed greater expression of fibrosis-related genes in BPH/2 mice, some of which could be reversed by antihypertensive treatment (Nelson et al., 2018).

To date, the impact of hypertension on the structure of the vasculature has only been assessed in cerebral arterioles. Baumbach and colleagues reported that BPH/2 mice exhibit cerebral arteriole hypertrophy but no reduction in external diameter (remodeling) such as that seen in other models of hypertension (Baumbach et al., 2003). This is supported by a preliminary abstract reporting hypertrophic inward remodeling in mesenteric arteries of BPH/2 mice (Jelinic et al., 2018). Recently, BPH/2 have been shown to develop retinal disease, with a thinner neural structure and complete loss by 21 weeks of age of the outer layers of the retina including the plexiform, nuclear, and photoreceptive layers (Herat et al., 2020). Functionally, BPH/2 mice are reported to have endothelial dysfunction in small caliber arteries, demonstrated by a 25–50% reduction in maximal vasorelaxation in response to acetylcholine and bradykinin (McGuire et al., 2007). Using Doppler ultrasound, BPH/2 have been shown to have impaired endothelium-dependent dilatation in femoral arteries (Nelson et al., 2018). A comprehensive myographical examination of arteries from different vascular beds of BPH/2 and BPN/3 found that the arteries from the hypertensive mice have greater myogenic tone and impaired relaxation despite having similar passive qualities (Tajada et al., 2012). In the same study using electrophysiology techniques, the authors found a decreased contribution of potassium ATP-dependent channels, which was also consistent with the findings of lower expression of some potassium channels including Kir2.1 and Kir4.1 (Tajada et al., 2012). Thus, there was a good correlation between the changes in excitability, the electrophysiological/pharmacological assessment of the contribution of potassium channels, and the molecular expression of specific channels in this study (Tajada et al., 2012).

Hypertensive BPH/2 mice are reported to release double the amount of hydrogen peroxide from the aorta compared with BPN/3, which may indicate a greater level of oxidative stress (Uddin et al., 2003b). Measurement of oxidants and antioxidants in the aorta showed that this may be attributable to elevated SOD activity and reduced catalase activity leading to greater production of hydrogen peroxide and decreased catalase-mediated conversion of hydrogen peroxide to water (Uddin et al., 2003b). Different expression of canonical transient receptor potential (TRPC) 3 channels and of hetero-multimeric TRPC channels may also contribute to differences in vascular tone in the BPH/2 mice (Alvarez-Miguel et al., 2017).

Hypertensive BPH/2 mice have similar kidney-to-body weight ratio as BPN/3 (Schlager et al., 1979) but have fewer nephrons per kidney (Rosenberg et al., 1982). Morphometric analysis of kidneys from older BPH/2 mice showed none of the characteristic features of renal parenchymal pathology (Rosenberg et al., 1979) indicating that major morphological differences in the kidney are not likely driving the hypertension. However, the available filtration surface area in juxtamedullary glomeruli in BPH/2 mice is less than half that of BPN/3 mice while the superficial cortical glomeruli were comparable between strains (Rosenberg et al., 1982). By contrast, it is the superficial glomeruli in BPH/2 mice that show reduced permeability of the basement membrane compared with BPN/3 mice but not the juxtamedullary glomeruli. Theoretically, reduced filtration surface and permeability could lead to decreased glomerular filtration rate, volume expansion, and hypertension (Rosenberg, 1983). However, glomerular filtration rate was shown to be less in juvenile BPH/2 mice than in mature BPH/2 mice (Rosenberg et al., 1985). Yet, both normotensive and hypertensive mice have comparable glomerular number until about 7 weeks of age. Glomerular number then plateaus in BPH/2 mice, which have markedly fewer glomeruli in adulthood compared with BPN/3 mice. Total body water, used as a measure of volume expansion, was also greater in younger BPH/2 mice than BPN/3 but this trend reversed with age and resulted in a lower total body water in older mice (Rosenberg et al., 1985). When a comparison is made between glomerular number and total body volume, it is noticeable that total body volume only starts to decrease toward normal levels after glomerular number becomes lower in the BPH/2 mice. Additionally, BPH/2 mice are not salt sensitive, suggesting natriuresis is not impaired (Leckie, 2001).

## Metabolic Abnormalities in BPH/2 Mice

An important phenotypic difference observed in BPH/2 mice is that their body weight is lower compared with BPN/3 mice (Rosenberg et al., 1982; Buu et al., 1987). However, hypotensive BPL/1 mice also have lower body weight than BPN/3 mice suggesting that this attribute is not likely to be associated with the hypertension (Rosenberg et al., 1979). Early studies suggested that water intake of female BPH/2 mice was 20% greater than the normotensive strain but if offered a choice, they preferred solutions of lower NaCl or KCl concentration (Bachmanov et al., 1998). The higher water intake was not observed in a later metabolic study of male mice in which it was 11% higher in BPH/2 mice, but the difference was not significant due to the small n value (Jackson et al., 2014b). Hypertensive BPH/2 mice are also markedly more active compared with BPN/3 mice, particularly during the dark phase of the daily light cycle, as shown by radio-telemetric measurement of locomotor activity (McGuire et al., 2007; Davern et al., 2009). While locomotor activity is positively associated with BP, hyperactivity alone is unlikely to contribute to the elevated BP. This is because analysis of covariance showed that elevated BP in BPH/2 mice compared with BPN/3 mice was independent of locomotor activity (Davern et al., 2009). Furthermore, normotensive C57Bl6 mice also have a similar level of activity as BPH/2 mice, indicating hyperactivity is not only apparent in hypertensive mice (Davern et al., 2009). Metabolic rate was greater in young BPH/2 compared to the normotensive control strain BPN/3 mice but was similar to normotensive C57Bl6 strain. When the activity of the mice was considered, there were no differences in energy metabolism per se (Jackson et al., 2014b). Also percentage lean mass was similar in young and older BPH/2 and BPN/3 suggesting that differences in body weight were due to lower fat mass in the more active BHP/2 (Jackson et al., 2014b).

The lower fat mass may also contribute to why hypertensive BPH/2 mice are less capable of thermoregulation than BPN/3 mice, as indicated by a greater rate of rise of body temperature, higher end temperature, and shorter time until death following exposure to 43°C heat (Malo et al., 1989). These differences may also indicate that thermoregulation may be abnormal in BPH/2 mice. However, the validity of these findings is questionable owing to exposure to heat taking place while the mice were anesthetized, causing thermoregulatory impairment (Kurz, 2001). However, Malo and colleagues also found that long-term (40 days) acute (5 min per day) exposure to 40°C heat could reversibly reduce the BP in BPH/2 mice by 20 mmHg, which was not due to the direct vasodilatory effect of the heat (Malo et al., 1990). One hypothesized cause of abnormal thermoregulation in BPH/2 mice is greater production of heat shock protein 70 (HSP70), which is overexpressed in the kidney of BPH/2 compared with BPN/3 mice following heat stress (Hamet et al., 1990). This suggests that BPH/2 mice may have abnormal cellular response to stress. However, it is also possible that central thermoregulatory control mechanisms may be different in BPH/2 mice. Indeed orexin, a central hormone known to be involved in thermoregulatory control, is overexpressed in hypothalamic tissue of BPH/2 mice as mentioned earlier (Marques et al., 2011a,b). Moreover, orexin is also involved in BP control, stress, sympathetic activity, and energy metabolism (Shirasaka et al., 1999, 2002; Szekely et al., 2002; Furlong et al., 2009), making it a potential mediator of many of the phenotypic abnormalities in these mice. Taken together, there are numerous metabolic abnormalities in BPH/2 mice and while some abnormalities such as thermoregulation appear to be related to BP, others such as locomotor activity appear to be independent of hypertension (Davern et al., 2009).

## BPH/2 Mice and Pregnancy, Atherosclerosis, and Diabetes

An additional subline of mice produced by Schlager and colleagues is the BPH/5 strain. Compared with C57Bl6 mice, this strain has moderately elevated BP, which is increased further during pregnancy (Davisson et al., 2002). As such, the BPH/5 strain has been used extensively as a model of pre-eclampsia (Butz and Davisson, 2001; Davisson et al., 2002; Cross, 2003; Dokras et al., 2006; Hoffmann et al., 2008). Treatment with the superoxide scavenger tempol during gestation improved fetal outcome and offspring survival and reduced the level of BP rise during pregnancy (Hoffmann et al., 2008). BPH/5 mice normally have half the number of pups than normotensive mice due to reabsorption of placentas, which was reduced by half with tempol (Hoffmann et al., 2008). The higher oxidative stress in mothers is consistent with many of the microarray studies that showed elevated expression of oxidative-stress-related markers in the BPH/2 mice (see above). Additionally, BPH/5J mice have greater adiposity, greater circulating leptin levels and leptin resistance, as indicated by the blunted effect of leptin on food intake and body weight (Sutton et al., 2017). Leptin resistance is a phenomenon also associated with sympathetically mediated BP elevations in other animal models (Prior et al., 2010, 2014); so leptin resistance may also contribute to the hypertension in this strain.

A more recent cross of BPH/2 with the apolipoprotein E-deficient mouse (APoE) was developed to examine the interaction between hypertension associated with high SNS activity with an atherosclerotic-prone mouse (Al-Sharea et al., 2019). These mice when fed a western diet developed unstable atheromatous plaques, the formation of which was independent of endothelial dysfunction. There were reduced levels of key hematopoietic stem and progenitor cells as well as sinusoidal endothelial cells and osteoblasts (Al-Sharea et al., 2019). Interestingly, all these effects could be reversed by a non-selective β-blocker propranolol.

Watson and colleagues recently examined the effect of streptozotocin-induced diabetes in the BPH/2 and BPN/3 strains (Watson et al., 2019). While diabetes did not change the degree of hypertension, the albuminuria was 3-fold higher in diabetic BPH/2 mice. This was most likely due to the hypertensive mice having greater levels of renal noradrenaline and dopamine and when combined with diabetes-induced abnormalities in levels of metabolic enzymes and impairment in antioxidant systems, produced greater overall oxidative stress (Watson et al., 2019).

## Perspective

**Figure 4:**
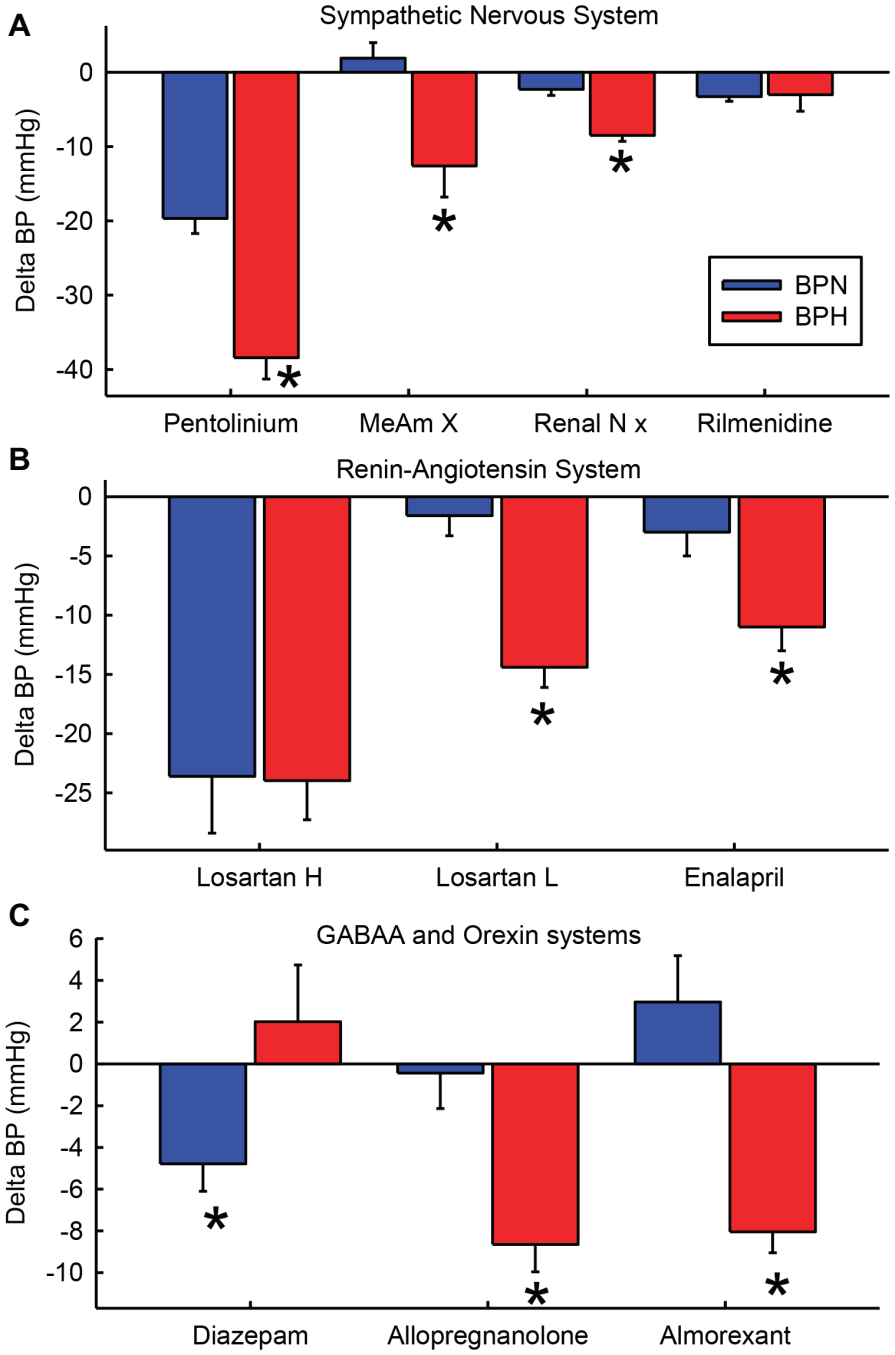
Average change in blood pressure (BP) after treatments that affect the sympathetic nervous system **(A)**, the renin-angiotensin system and kidney **(B)** and GABAA and orexin systems **(C)** in BPN/3 (blue) and BPH/2 (red) mice. Treatments are pentolinium (Davern et al., 2009), medial amygdala lesions [MeAm X (Jackson et al., 2014d)], renal nerve denervation [Renal N x (Gueguen et al., 2019)], rilmenidine (Jackson et al., 2014c), high-dose Losartan [Losartan H 150 mg/kg/day (Palma-Rigo et al., 2011)], low-dose Losartan [Losartan L, 8 mg/kg/day (Jackson et al., 2018)], enalaprilat [1.5 mg/kg (Jackson et al., 2013)], diazepam [2.5 mg/kg/day orally for 7 days (Davern et al., 2014)], allopregnanolone [s.c. 5 mg/kg/day for 14 days (Stevenson et al., 2017)], almorexant [30 mg/kg i.p. (Jackson et al., 2016)]. *Indicates *p* < 0.05 between strains.

The development of the strains of hypertensive mice by Gunther Schlager and colleagues in the 1970s has led to a comprehensive examination of mechanisms of hypertension. This led to the discovery that the hypertension is principally related to chronic activation of the SNS and as such is one of the few true models of neurogenic hypertension (Figure 4A). Our own research has revealed that a major mechanism involves GABA_A_R dysfunction within the CNS and most likely within the amygdala and hypothalamus (Figure 4C). Neurosteroids such as allopregnanolone offer a possible new opportunity to treat the neurogenic contribution to human hypertension. Importantly, the site of action is quite distinct from that of benzodiazepines like diazepam (Figure 4C). Exciting new discoveries that orexin systems are also involved come from the selective antihypertensive effect of the orexin receptor antagonist almorexant (Figure 4C). However, despite some evidence from animal models of hypertension, the role of limbic brain regions seems to be relatively unrecognized in human hypertension. The currently available centrally acting antihypertensive agents such as moxonidine or rilmenidine act predominantly in the brainstem and this is where interest seems to have remained in the clinical setting. However, these drugs are ineffective in BPH/2 (Figure 4A).

To date, the evidence for the involvement of limbic regions in human essential hypertension is limited to indirect associations. For instance, using blood-oxygen-level-dependent functional magnetic resonance imaging, greater amygdala activity is associated with greater BP reactivity to stress, which in turn is associated with greater risk of developing hypertension (Ming et al., 2004; Gianaros et al., 2008). Importantly, the Schlager strains have led us to understand that the SNS also influences mechanisms within the kidney, controlling renin, the vasculature and oxidative stress as well as other systems including the immune system and metabolic effects. Low doses of RAS blockers are much more effective in BPH/2 than in BPN/3 mice as is renal denervation, suggesting an important interaction between the brain and the kidney (Figure 4B). Thus, hypertension is one manifestation of SNS overactivity but the interactions of the SNS with other systems have far-reaching implications. The data may have implications for the effects of psychosocial stress on health generally.

## Author Contributions

All authors contributed to the drafting, editing and finalizing of the review.

### Conflict of Interest

The authors declare that the research was conducted in the absence of any commercial or financial relationships that could be construed as a potential conflict of interest.
